# Exploring patients’ and carers’ experiences, understandings and expectations of COPD exacerbations:an interview study

**DOI:** 10.3399/BJGPO.2024.0026

**Published:** 2025-05-07

**Authors:** Ann Hutchinson, Richard Russell, Helena Cummings, Omar Usmani, Sarah MacFadyen, Judith Cohen, Tamsin Morris, Hana Muellerova, Yang Xu, Gary Hellens, Kay Roy, Michael G Crooks

**Affiliations:** 1 Hull York Medical School, University of Hull, Hull, UK; 2 Peter Gorer Deptartment of Immunobiology, King’s College London, London, UK; 3 Hull University Teaching Hospitals NHS Trust, Hull, UK; 4 Imperial College London, London, UK; 5 Asthma and Lung UK, London, UK; 6 Hull Health Trials Unit, University of Hull, Hull, UK; 7 AstraZeneca UK, London, UK; 8 AstraZeneca BioPharmaceuticals Medical, Cambridge, UK; 9 University College London Hospitals NHS Foundation Trust, London, UK

**Keywords:** COPD, exacerbations, breathlessness, general practitioners

## Abstract

**Background:**

Chronic obstructive pulmonary disease (COPD) exacerbations are clinically significant events that affect millions of people globally.

**Aim:**

To explore patients’ and carers’ experiences, understanding, and expectations of, as well as their responses to, exacerbations.

**Design & setting:**

Semi-structured interviews conducted with patients who have COPD and their carers from four sites across England.

**Method:**

Interviews were conducted with a purposive sample of patients with COPD and their carers recruited from four sites in England: two in Yorkshire, one in Hampshire and one in London. Interviews were theoretically informed by the Breathing Space concept and analysed using reflexive thematic analysis. This research is reported in line with the Standards for Reporting Qualitative Research.

**Results:**

Forty patient participants were recruited: 21 were female, 28 were White, with a mean age 69 years (standard deviation [SD] = 8.1 years), mean COPD duration = 11.3 years (SD = 8.3 years), median exacerbations in past year = 1.5 (range 0–9). Seven carer participants were recruited; of these, six were female and six were White. Three themes were identified: the language that clinicians use in COPD is important; episodes of symptom worsening have profound impacts on patients and carers; and patients’ early experiences, including the responses of clinicians to their help-seeking, have a lasting effect on their behaviour. How patients respond to symptom worsening can be considered holistically in the context of the Breathing Space framework. Breathlessness affected all patient participants and was a key symptom that precipitated action.

**Conclusions:**

Our findings show how early help-seeking experiences shape later behaviour. Early emphasis on symptom management, preparation for exacerbations, and post-exacerbation reviews are practical ways that clinicians can support patients and carers to manage these events better. The Breathing Space concept provides a useful framework to identify needs and tailor COPD management appropriately.

## How this fits in

Most patients don’t understand the term ‘exacerbation’, but exacerbations have a significant impact on their wellbeing and the disease progression. This research shows that patients prefer clinicians to use plain language or the term ‘flare-up’ when discussing exacerbations, and that they want to be told early on that their symptoms may worsen and what to do about it. The language used when discussing symptom worsening is important to enable good communication between patients, carers, and clinicians. How patients are responded to by clinicians when they first seek help for worsened symptoms has a long-lasting effect on their later help-seeking behaviour, so it is important to offer symptom management alongside disease treatment from the outset.

## Introduction

Chronic obstructive pulmonary disease (COPD) is a common lung disease that has significant associated morbidity and mortality.^1^ Symptoms (for example, breathlessness, cough, and sputum production) typically worsen over time, and are punctuated by episodes of rapid worsening, termed ‘exacerbations’. Exacerbations are significant events^2^ and associated with worse quality of life, airway inflammation, lung-function decline, and increased mortality risk.^3–5^ Exacerbations are often classified as mild, moderate, or severe, based on medication and healthcare use.^6^ It is recognised that mild exacerbations recorded using symptom diaries are more frequent than those identified through treatment/healthcare resource utilisation^6^ and, despite being termed ‘mild’, contribute to symptom progression.^7^

Exacerbations elicit an intensely emotional response and greatly affect patients’ daily activities, leading to them feeling uncertain over what actions they should take and their feeling in need of ‘rescue’.^2,8,9^ Discrete choice experiments^10^ and interviews^9,11,12^ have revealed that patients find the limitations on daily life that result from exacerbations to be troublesome. Although they are aware that their symptoms change before an exacerbation, most don’t recognise that this signals an impending exacerbation. Exacerbations are very individual experiences in terms of symptoms experienced and resultant help-seeking.^2,10,11^

Linnell and Hurst^2^ suggested that patients find the word ‘exacerbation’ complicated and, instead, advocate the use of lay language. Other terms that are more evocative of the severity of these events and readily translatable around the world, such as ‘crisis’, have also been proposed.^13^

**Figure 1.**
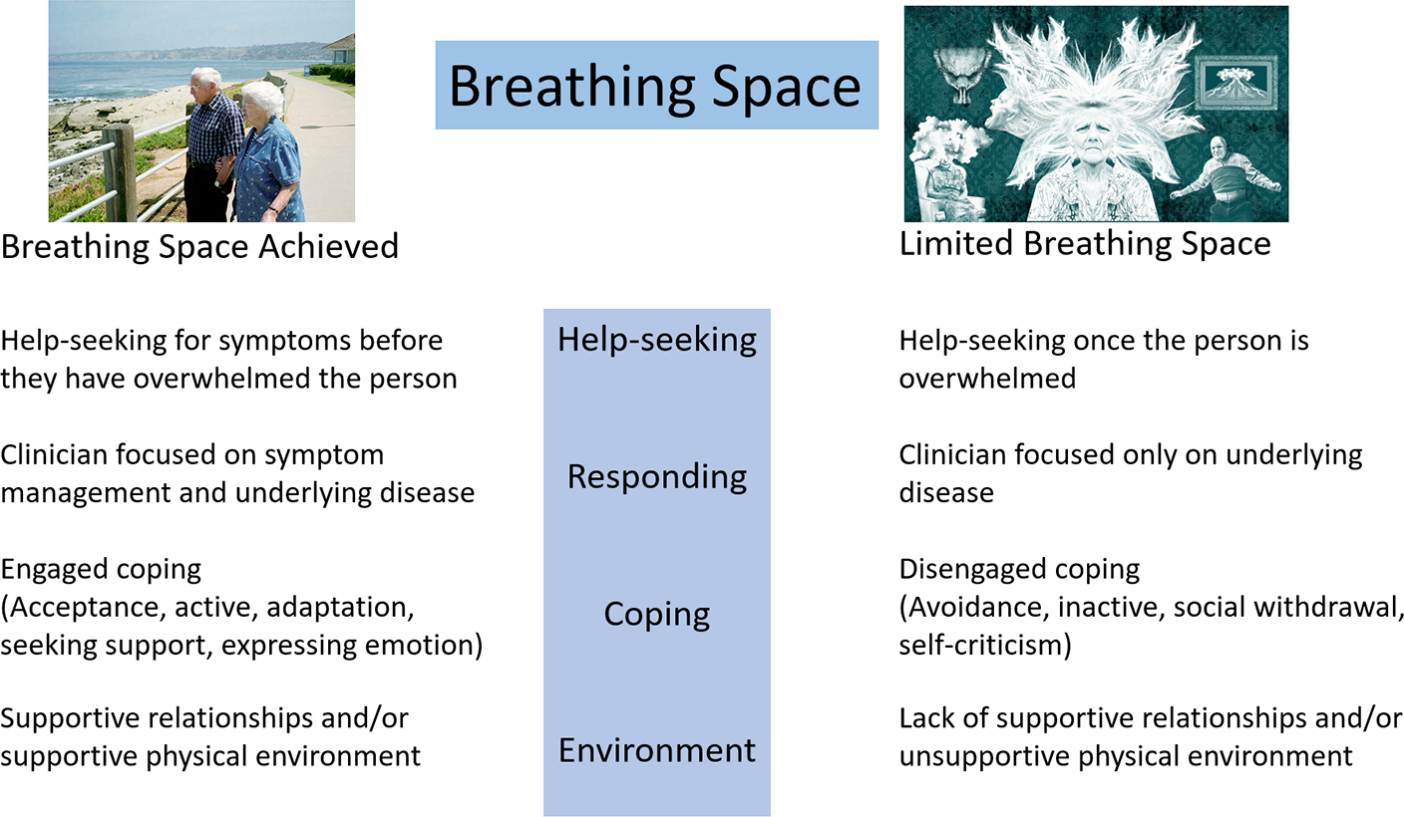
The Breathing Space concept

Breathing Space^14^ (Figure 1) is a concept developed from a systematic literature review and qualitative synthesis on the experience of breathlessness. It conceptualises how to live well with chronic breathlessness, with the degree of ‘breathing space’ a person can achieve being related to several interacting factors:

- how the person seeks help (that is, in crisis or not),
- how they are responded to by clinicians when they seek help, and
- how they cope with their symptoms (that is, in an engaged or disengaged fashion).

This concept can be applied beyond breathlessness, and has the potential to be used as a framework to understand how people with COPD respond to periods of symptom worsening.

In this study we explored patients’ and carers’ experiences, understanding, and expectations of exacerbations. Additionally, we investigated how patients responded to periods of symptom worsening and how their early experiences of exacerbations affected their subsequent behaviour. The research was reported in line with the Standards for Reporting Qualitative Research.^15^

## Method

We undertook a multicentre, semi-structured interview study. Eligibility criteria are outlined in Table 1. Participants were purposively selected for maximum variation — in terms of gender, urban/rural location, ethnicity, prone/less prone to exacerbations — from four sites in England: two in Yorkshire, one in Hampshire, and one in London.

**Table 1.** Inclusion and exclusion criteria

**Inclusion criteria: patients** – Aged ≥30 years – Clinician-diagnosed COPD – Documented airflow obstruction on spirometry (FEV-1/FVC ratio<0.7) on record – Current or ex-smokers with at least 10 pack-year smoking history – At least one previous self-reported ‘worsening’ or ‘exacerbation’ of COPD symptoms – Access to a telephone – Willing and able to participate in a semi-structured interview conducted in English – Group A (prone to moderate/severe exacerbations) At least two moderate^a^ and/or one severe^b^ exacerbation of COPD within 1 year before 31 December 2019; and/or At least two moderate and/or one severe COPD exacerbation since 1 January 2020 – Group B (less prone to exacerbations) No more than one moderate^a^ and no severe^b^ exacerbations of COPD within 1 year before 31 December 2019; and No more than one moderate and no severe exacerbations of COPD since 1 January 2020 **Inclusion criteria: carers** – Carer for a patient in the study – Access to a telephone – Willing and able to participate in a semi-structured interview conducted in English **Exclusion criteria: patients and carers** – Unwilling or unable to consent or complete study measures

^a^A moderate exacerbation of COPD is defined as a worsening of symptoms and/or decline in lung function that results in treatment with oral antibiotics and/or oral corticosteroids without hospital admission or emergency department attendance. ^b^A severe exacerbation of COPD is defined as a worsening of symptoms and/or decline in lung function that results in hospital admission or emergency department attendance.

COPD = chronic obstructive pulmonary disease; FEV-1 = forced expiratory volume in 1 second; FVC = forced vital capacity;

Eligible patients and carers were identified by their usual care team. An explanation about the study was provided and those expressing an interest were given written information and an opportunity to ask questions prior to providing written informed consent. Demographic and clinical data were collected by site researchers prior to interviews being conducted by a qualitative researcher.

### Data collection

#### Demographic and clinical characteristics

Data were obtained for patient participants via self-reporting and review of their electronic health record, and included: age, gender, ethnic group, COPD history, treatment history, healthcare utilisation, spirometry, modified Medical Research Council breathlessness score, COPD Assessment Test score, and number/nature of exacerbations in 2019 and the 12 months before participation. Exacerbation data were recorded in this way in recognition of the impact the COVID-19 pandemic had on exacerbations. The first wave of the pandemic in the UK began in March 2020. Data are reported descriptively.

#### Interviews

Interviews were conducted by telephone to minimise physical contact during the pandemic and consent was reconfirmed at the beginning of each interview. Taking a phenomenological approach,^16^ in-depth, semi-structured interviews followed a topic guide (Table 2), which was developed by the research team based on a targeted literature review and through discussion with members of the ACT on COPD steering committee. Patients and carers were interviewed together when possible, but separately if necessary. Interviews were audiorecorded, transcribed verbatim by an independent transcriber, and checked for accuracy by the interviewer.

**Table 2.** Topic guide

Question
What symptoms do you experience as part of having COPD?
Do they get much worse sometimes, or do they stay roughly the same?
What does the word ‘exacerbation’ mean to you?
How would you describe the term exacerbation in your own words?
Can you tell me about the first time you remember having an exacerbation (use patient’s own term)?
How do you recognise that you have an exacerbation (use patient’s own term) coming on?
What do you do if you feel an exacerbation (use patient’s own term) coming on?
Do you sometimes feel your symptoms have worsened and not seek medical help?
How do exacerbations (use patient’s own term) affect your health?
What do the terms ‘mild exacerbation’, ‘moderate exacerbation’, and ‘severe exacerbation’ mean to you?
How has the COVID-19 pandemic affected your health?
What will you do the next time you have an exacerbation?
If I asked you to draw what an exacerbation (use patient’s own term) feels like to you, what would it look like?

COPD = chronic obstructive pulmonary disease;

### Data analysis

Reflexive thematic analysis^17^ was selected as a flexible method that could be underpinned by a phenomenological perspective^16^ to gain a rich understanding of the meaning of participants’ experiences, understandings, and expectations of exacerbations. Analysis involved: i) immersion in the data; ii) line-by-line production of descriptive labels (codes); iii) clustering of descriptions around meanings that arose from the participants, which was done to identify themes; iv) reflection on each theme by the research team while attending to the deeper existential issues experienced by participants in addition to practicalities and contextual details; and v) reflection on each theme in light of the whole data and Breathing Space. The data were managed using NVivo (version 12).

## Results

### Participant characteristics

Participants were recruited at four sites (three in secondary care, one in primary care) between February and August 2021. In total, 44 patients consented to participate, although four withdrew prior to interview. Forty patient participants were recruited, of whom 21 were female, 28 were White, and 23 were exacerbation prone. Their mean age was 69 years (standard deviation [SD] = 8.1 years), the mean COPD duration was 11.3 years (SD = 8.3 years), and the median number of exacerbations in the previous year was 1.5 (range 0–9) (Supplementary Table 1). Seven carer participants were also recruited; of these, six were female and one male, and six were White and one Asian. All patient participants were interviewed — seven together with their carer — and contributed data to the analysis.

Recruitment was balanced between study sites (Hull *n* = 10, East Riding of Yorkshire *n* = 9, London *n* = 12, and Hampshire *n* = 9), with both urban and rural settings represented. Interviews lasted for a mean duration of 43 (range 15–97) minutes.

### Identified themes

Three themes were identified:

- the language clinicians use in COPD is important,
- episodes of symptom worsening (exacerbations) have a profound impact on patients and carers, and
- patients’ early experiences, including the responses of clinicians to their help-seeking, have a lasting effect on their behaviour.

These are described below with verbatim quotations (P = patient, C = carer); additional quotations are given in Supplementary Table 2.

#### The language clinicians use in COPD is important

Approximately half of participants reported that the term ‘exacerbation’ meant nothing to them and, although the other half recognised that it refers to episodes during which they get worse, very few used the word and many reported finding it difficult to say:

*‘Exac, exac, exaper…I can’t even say it now…’* P1

Participants were more likely to use plain language than specific words to define episodes of symptom worsening. Indeed, when asked which terms they would use in place of exacerbation (for example, ‘flare-up’, ‘crisis’, ‘worsening’, or ‘lung attack’), many participants still preferred to use their own words to describe the symptom(s) that they experience. However, of the proposed terms, ‘flare-up’ was most commonly chosen, with some choosing ‘attack’. Although a few proposed the term ‘chest infection’, none chose ‘crisis’ or ‘worsening’. The term used to describe an exacerbation appeared to alter the threshold at which an individual would perceive that they were experiencing it:

*‘I would see a flare-up as a milder description than an attack, something short lived and it doesn’t make you think you’re going to die and* [result in your] *dialling 999.”* P26

The terms ‘mild’, ‘moderate’, and ‘severe’ exacerbation (clinical definitions based on medicine and/or healthcare use) were meaningless for the majority of patients and none used these terms. When asked to characterise mild, moderate, and severe exacerbations, most could not; those who did based their definition on symptom severity and impact, rather than the required/received treatment:

*‘I don’t think I do mild to moderate to severe, I think I just go from nothing to really bad.’* P25

#### Episodes of symptom worsening (exacerbations) have a profound impact on patients and carers

All participants reported increased breathlessness at the time of exacerbation and this was the symptom that most commonly prompted a change in patient behaviour and/or help-seeking:

*‘Being out of breath is the main one; that can strike yer at any time.’* P2

The rate of breathlessness onset varied between individuals.

Other commonly reported symptoms at the time of exacerbation included fatigue, cough, chest tightness, and sputum production/colour change. Some also reported poor sleep and swollen legs. Symptoms often interacted with each other, thereby increasing their impact, and participants described associated feelings of depression, worry, fear, panic, and a loss of control:

*‘I’m getting more and more depressed, to be honest. I wouldn’t never harm meself, but it’s getting to the state where I wish I weren’t here.’* P14

*‘I don’t really speak about it...I don’t want to burden ‘em* [other people]*, with my problems.’* P36

Participants with more experience of exacerbations were better able to recognise and describe their own pattern of symptom worsening than those participants who were less exacerbation prone.

The speed at which symptoms developed/worsened exhibited high interindividual variability and affected participants’ ability to identify exacerbation onset. Some described onset as being very sudden:

*‘It was totally out the blue, when I couldn’t catch me breath.’* P40

Many others described a gradual symptom build-up, potentially representing an identifiable exacerbation prodrome. However, gradual symptom onset was a potential barrier to self-awareness:

*‘It sorta creeps up on me; it’s so gradual that you don’t notice it happening. Probably a week to 2 weeks…. Then it’s reached that point where it’s noticeable and you think, “hang on, my breathing is not good”.’* P1

**Figure 2.**
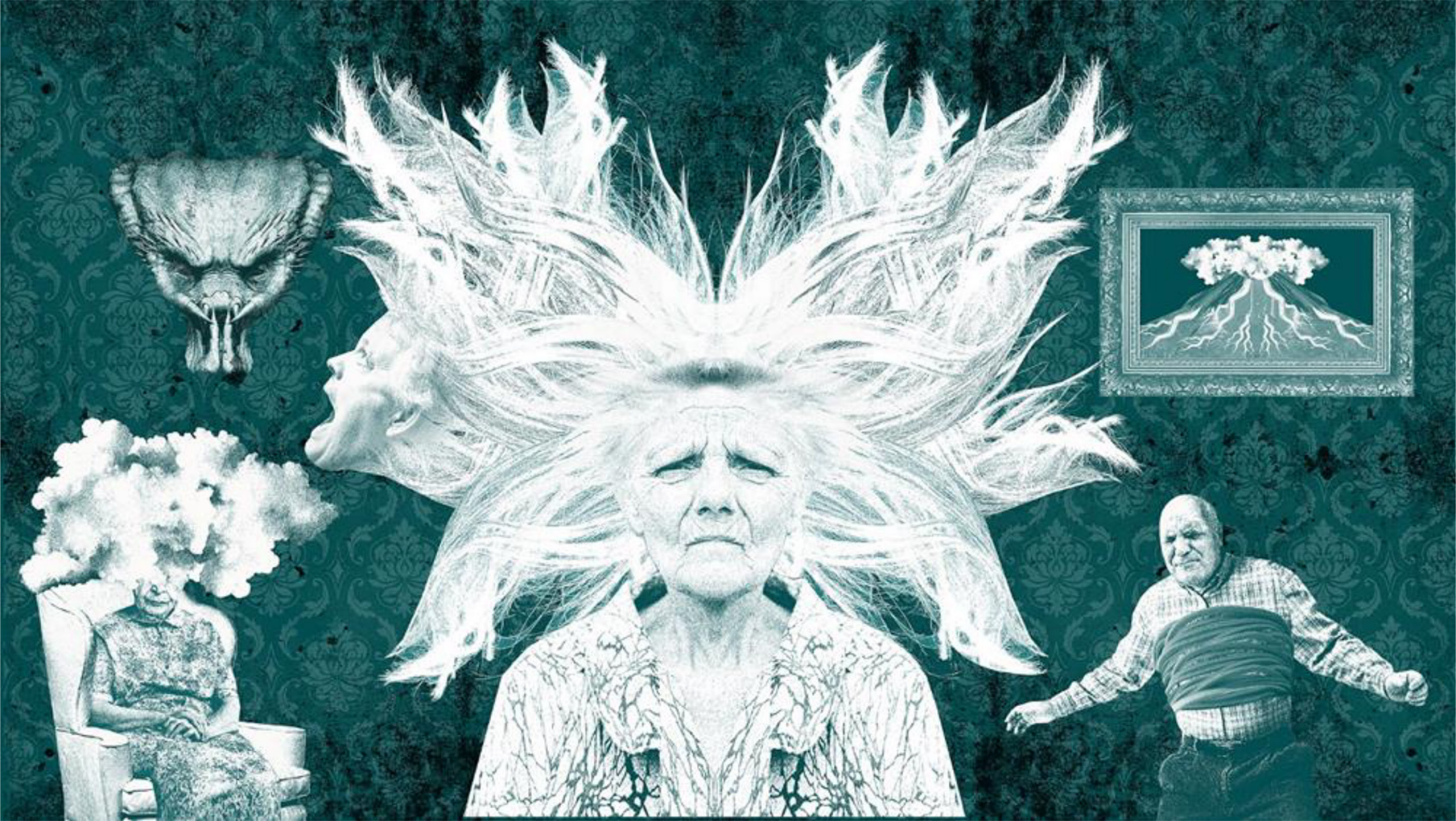
Artist’s impression of how it feels to have an exacerbation developed from descriptions given by participants detailed in Supplementary Table 2. (Credit: Bluebeany, for the Understanding People’s Experiences of Exacerbations (the ExacQual Study), Wolfson Centre for Palliative Care, Hull York Medical School)

Participants’ vivid descriptions of their exacerbation experiences are given in Supplementary Table 2. Artwork representing their experience is shown in Figure 2.

Many participants described identifiable triggers that worsened their symptoms, including the weather, pollen, pollution, smoke, scents, stress, lifting, going up stairs, talking, and infections:

*‘It can vary day to day. Extremely hot weather, forget it, I can’t do anything. Cold weather, I just can’t tolerate that at all. And strong-scented things, they bring it on.’* P15

Others were not able to identify anything that precipitated symptom worsening:

*‘I can’t think of anything that triggers it. It’s just normal and then much worse. I haven’t noticed anything, just that it comes on on its own.’* P2

Some participants recognised persistent/progressive functional limitation and general health decline following exacerbations, and also noticed increased exacerbation frequency and prolonged recovery over time:

*‘You have a flare-up, you sit in a chair and you wait for the next one... my health is nowhere near what it used to be.’* P9

*‘Every year I have more flare-ups than the year before.’* P7

However, some didn’t notice any long-term impact and described making a full recovery:

*‘It’s a short-term effect, then back to normal.’* P30

Carer participants reported being prepared to look after their family members when their symptoms got worse, but described a negative impact on their own wellbeing as a result of feelings of worry, helplessness, stress, and frustration:

*‘You hear him through the night if he’s struggling with his breathing, and it tends to disrupt your sleep.’* C17

*‘Once she starts coughing she coughs for quite a long time, and that’s always worrying.’* C33

*‘It makes me feel frustrated because I know there aren’t exact answers to these questions but some more guidance from the doctor would be helpful.’* C16

Carer participants also described becoming vigilant to changes in symptoms and being unable to plan activities.

#### Patients’ early experiences, including the responses of clinicians to their help-seeking, have a lasting effect on their behaviour

Most participants reported feeling unprepared for their first COPD exacerbation and none recalled making individualised self-management plans with their clinicians at any point:

*‘I did not expect it* [exacerbation]*; I just expected to slowly get worse.’* P16

Only those with family members who had been diagnosed with COPD, or those who had attended pulmonary rehabilitation before having exacerbations, knew what to expect:

*‘I’m well up on COPD because I’ve got family history of it.’* P4

*‘That is the best thing I ever did was go to pulmonary rehab. I learnt everything I needed to know.’* P13

For many, their first exacerbation was the event that led to their diagnosis and, even those with a prior COPD diagnosis, reported having never been told about exacerbations. The consequence of the lack of awareness that an exacerbation might occur was delayed recognition and treatment, leading some to be hospitalised, often in frightening circumstances:

*‘For about 2 weeks I’d been struggling with my breathing…and then one day I just hit the floor, couldn’t breathe. They rushed me into* [the emergency department]*. I wasn’t prepared for hospital, no.’* P15

*‘I was at home and my breathing had got worse during the course of the day…when the doctor came, he said, “you’re having an exacerbation and you need to be in the hospital”. That was very frightening! The only thing that I wished had been different was that I had been better informed to expect this sort of thing happening.’* P2

Although participants’ day-to-day experiences of COPD and the rate of symptom change at exacerbation onset both influence help-seeking behaviour, clinician responsiveness to early help-seeking appears to be a very important determinant. Many participants perceived that they were only offered medical treatment for COPD, with no focus on non-pharmacological symptom management:

*‘Nobody seems to be explaining to me really why me breathing’s got as bad as it has.’* P14

*‘I was never given any specific advice. I was given this inhaler and they said, “Take it whenever it’s required” and that was it.’* P32

Consequently, their ability to achieve Breathing Space became restricted. These participants were likely to describe a reliance on inhaled medication:

*‘I take me inhaler. There isn’t a lot more I can do.’* P14

Often patients described using their inhaler beyond prescribed doses and some participants who were not taught symptom management strategies by their clinicians relied heavily on repeated use of ‘rescue packs’ (that is, antibiotics and/or steroids) without review:

*‘I saw the specialist in January and he checked me records back and I’d had nine courses of steroids in that year.’* P7

Others with restricted breathing space were likely to present directly to hospital:

*‘When I very first got this, I went backwards and forwards to the GP. The GP diagnosed me with nothing! Then, I couldn’t even lie down, I’ve never felt as ill as that! And I just said: “Do you know what, we’ll have to get an ambulance cos I’ve had enough.” And I spent a fortnight laying in hospital.’* P25

Several participants who perceived that their clinician failed to acknowledge the impact their symptoms had on them developed a reluctance to seek help for worsened symptoms:

*‘I think maybe, on occasion, I should have got help, but I feel guilty every time I ring the doctors up; I feel like they think, “Oh God, there’s nowt wrong with her, she’s just a bit breathless, what’s she bothering us for?”’* P1

In contrast to these participants, many others were able to achieve Breathing Space as they had clinicians who offered continuity of care and non-pharmacological approaches to symptom management, in addition to medication:

*‘I have been seeing her* [GP] *for nearly 11 years now, she knows my ins and outs. She’s the one that put me up for* [pulmonary rehabilitation]*…I also talked to the nurse about the fact that my husband and I didn’t have a love life anymore. She gave me some helpful tips to do things with each other without causing anxiety or breathlessness to me as well.’* P23

Encouraged by their clinicians, several participants demonstrated engaged coping^18^ by adopting behaviours to improve their overall health, including stopping smoking, being more active, and losing weight:

*‘I mean I had to keep active anyway because if I don’t it’d only start messing with me chest. The more I keep active, the better.’* P13

However, this was not always straightforward, due to loneliness and a lack of effective support:

*‘I mean the NHS stop-smoking services really don’t do much for me at all. No, it’s just too simplistic. I think, for me, smoking is a lot more complex than that.’* P31

Many had learned engaged coping strategies from their clinicians to help them manage their symptoms, including pacing activities, accepting limitations, breathing techniques, and seeking support:

*‘I’ve become more active since I did pulmonary rehab classes. When it first happened I stopped doing stuff; now I don’t. I go up the stairs when I’m at work and, yeah, I am out of breath at the top, but I know it’s not hurting me, it’s doing me good.’* P1

However, comorbidities frequently affected patients’ ability to cope in an engaged fashion:

*‘They asked that I try* [pulmonary rehabilitation] *again, but I have a problem with that as I have a lot of knee pain.’* P29

*‘I’ve got mental health problems and I find it difficult leaving the house. I can’t just casually go out for a walk.’* P33

Participants who perceived that their clinician understood the impact of symptoms on their life, and who had been offered symptom-focused, non-pharmacological interventions early on in the course of their disease, were more likely to report adopting a stepwise approach to self-management. This included making use of non-pharmacological self-management strategies — like distraction, relaxing, and using breathing techniques — before resorting to using a rescue pack or contacting a clinician:

*‘The breathlessness; that’s the one. When that gets bad I would use the fan and I would use the nebuliser more frequently, and I would monitor my progress, and then contact the GP to see if I needed the steroids.’* P2

Some of these participants attended hospital based on advice from a doctor or previous experience that it was necessary:

*‘I lay down and try to relax me full body. Then I say, “Come on get your head together” and then I start relaxing. When I cannot get my breath and I’ve been on me inhaler and me nebuliser and still nothing, I’d go ahead and call an ambulance cos I know I’m bad.’* P11

Access to rescue packs had perceived advantages for those who were confident in their ability to self-manage their symptoms, and provided a sense of security and control as patients knew there would be no delay in starting medication due to difficulties getting an appointment with a health professional:

*‘Oh, it makes me feel quite secure…but I mean I wouldn’t wanna take steroids too often.’* P31

*‘I think it gives me a lot of confidence and the confidence leads to you to think, “I* am *controlling it”. I* am *aware of my condition, and so that helps me to control my life.’* P8

Those participants confident in their ability to self-manage described often trying a variety of other measures before resorting to using a rescue pack.

## Discussion

### Summary

These findings show the importance of using plain language and preparing patients for symptom worsening. and demonstrate that a clinician’s response to help-seeking for exacerbations can have lasting effects on patient behaviour.

### Strengths and limitations

This study had a large and diverse sample of patients. The sampling method ensured wide variation in how exacerbation prone patients were, and in their geographical location and ethnic group. However, not all patient participants had carers and some declined consent for their carers to be contacted, so the diversity of carer participants is limited. Only English-speaking participants from a single country with a national health service were included in this study; this limits the generalisability of our findings to other countries and health systems.

### Comparison with existing literature

In line with those of other studies,^2,19,20^ the participants in ours found the word ‘exacerbation’ difficult to say and understand. They preferred using plain language to describe symptom worsening, with ‘flare-up’ being the preferred term for such events; this aligns with the literature in which the term ‘flare-up’ has been reported to be that preferred by nurses^20^ and patients responding to an international survey.^21^ Very few participants expressed a preference for the term ‘attack’ or ‘chest infection’, and none chose ‘crisis’, in contrast to other studies.^19,21^

Our findings suggest that the term that is used affects the threshold at which an individual may consider themselves to be experiencing an exacerbation. It is important to balance the benefits of using striking terms, such as ‘attack’ or ‘crisis’, with the risk that such terminology may lead to underreporting. The term ‘flare-up’ may be a more-balanced term, with the potential to represent the full spectrum of exacerbation severities. ‘Flare-up’ is also used in other long-term conditions, such as rheumatoid arthritis, where treatment focuses on early disease modification in order to avoid long-term disability, a treatment paradigm that is desirable in COPD.

Worsening breathlessness was experienced by all patient participants, and various other symptoms were described, as seen in previous studies.^8,9,19,22^ Patient participants described individual patterns of symptom change and, in contrast to earlier findings,^9,19^ those with experience of exacerbations were aware of, and responded to, the pattern of symptoms that indicated exacerbation onset. However, those with less experience described difficulty differentiating day-to-day symptom variation from exacerbation onset.

The impact of symptoms was widespread both physically and psychologically, as has been reported elsewhere.^8,9,14,19,23^ A conceptual model^24^ of anxiety development in patients with COPD shows that many aspects of COPD induce anxiety, including experience of the first exacerbation. COPD diagnosis, changes to societal roles, and stigma also contribute to anxiety, often compounded by avoidance of physical activity and reliance on hospital care, rather than self-management.

Exacerbations can have a marked effect on carers as well as patients, and carers in this study reported that looking after their family member was not without difficulty; this has also been reported elsewhere.^11,19,25,26^ Increased vigilance was common, paralleling the ‘watchful protection’ described by Harrison *et al*.^8^ Our study highlighted the impact of exacerbations on the lives of carers, including their inability to plan, and limitations in terms of, activities.

The Breathing Space^14^ concept, which was first developed to describe the experience of chronic breathlessness, also has explanatory power with respect to how people respond to exacerbations. We found that most participants were unprepared for their first exacerbation and, for some, it was a traumatic experience, leading to lowered trust in clinicians. To our knowledge, our study is the first to describe how a patient’s early experience of their clinician’s response to their seeking help impacted the development of subsequent coping strategies, help-seeking behaviour, and the degree of Breathing Space^14^ achieved. Many perceived that taking medication and/or emergency presentation were all that could be done when their symptoms worsened; in contrast, other participants, whose clinicians offered a more-holistic approach, developed engaged coping strategies and were, therefore, able to manage their exacerbations better and achieve greater Breathing Space. Our findings show how early guidance in using non-pharmacological symptom management techniques may empower self-management and, potentially, decrease the likelihood of emergency presentations. The Breathing Space concept is in accordance with the conceptual framework of COPD-related anxiety developed by Christiansen *et al,* ^24^ highlighting the detrimental effects that breathlessness-induced fear, activity avoidance, and reliance on hospital attendance in crisis all have in terms of limiting patients’ ability to live well with COPD.

Although some patients with restricted Breathing Space reported overusing rescue packs, with a subsequent risk of long-term, treatment-related side-effects, we found that patients with a good knowledge of non-pharmacological symptom management and an understanding of their own symptom pattern/variation felt that they benefited from access to these packs. Such patients often had an awareness of the possible medication-related adverse effects and only resorted to using the packs when non-pharmacological strategies and increased reliever inhaler use had failed to provide a resolution; this finding is in accordance with the findings of Laue *et al*.^27^ Additionally, patients valued the guidance of clinicians when deciding to take rescue packs and what to do next if their symptoms did not improve with treatment; therefore, when considering the use of rescue packs in practice, their use should be seen as an addition to the care of clinicians and not a replacement for it, and patient selection is critical.

Not all participants were aware of the long-term effect of exacerbations on their health, only some had reviews with their clinician after an exacerbation and none had an individualised breathlessness management plan, despite these being recommended in guidelines from the National Institute for Health and Care Excellence (NICE)^28^ and NHS England’s breathlessness pathway.^29^ This finding is consistent with a British Lung Foundation report,^30^ which stated that the majority of patients with COPD were not receiving the five fundamentals of care set out by NICE. Overall, the implications of our findings are commensurate with the quality standard position statements drawn up by Bhutani *et al,* ^31^ which emphasise timely diagnosis, adequate patient and carer education, offers of non-pharmacological symptom management, appropriate management of exacerbations, and regular reviews of individualised care plans.

### Implications for research and practice

Recommendations for future research include:

- conducting a survey on how patients differentiate day-to-day variation from an exacerbation,
- devising and testing a needs assessment tool based on the Breathing Space concept,
- studying how best to implement NICE guidelines in practice, and
- developing and testing an educational intervention to prepare patients for exacerbations.

In terms of clinical practice, we recommend that clinicians use plain language — for example, ‘when your symptoms get worse’ or the term ‘flare-up’ — when discussing symptom worsening with patients and carers, instead of using the word ‘exacerbation’. Clinicians also need to be aware of, and show understanding about, the widespread physical and psychological impacts that exacerbations can have on patients and carers. To avoid potentially traumatic first experiences of exacerbations, clinicians should consider early discussion with their patients that their symptoms might worsen and what to do if this happens. Additionally, patients should be empowered to seek support when such events occur and reviewed to consider how to prevent/delay further exacerbations.

As some patients have difficulty recognising their individual symptom pattern, we recommend that clinicians guide them, and their carers, on how to recognise exacerbations and the correct actions to take in such circumstances. In order to support patients to respond appropriately to their symptoms, it is important that clinicians offer them both pharmacological and non-pharmacological management; this, combined with the co-creation of a personalised self-management action plan, may help patients to achieve Breathing Space and reduce overreliance on rescue medications and future crises.

The Breathing Space concept provides a useful framework that may help clinicians and patients to identify their current coping strategies and what behaviours they have learnt from early responses to help-seeking. This, in turn, may help to ensure their COPD management is tailored appropriately. Although rescue packs may be useful for those who are equipped to use them correctly and have ready access to clinicians for support, care should be taken for those with restricted Breathing Space and less access to timely clinician advice (where overuse may be more likely). We recommend that each exacerbation be viewed as an opportunity to prevent subsequent exacerbations, and that patients are reviewed so management can be optimised.

## Data

The dataset relied on in this article is available from the corresponding author on reasonable request.
